# Giant Left Ventricular Thrombus Formation in the Setting of Severe Aortic Stenosis and Heavy Cigarette Smoking

**DOI:** 10.7759/cureus.9130

**Published:** 2020-07-11

**Authors:** Mohamed Elmassry, M Rubayat Rahman, Timothy Dixon, Brandon Rogers, Nandini Nair

**Affiliations:** 1 Internal Medicine, Texas Tech University Health Sciences Center, Lubbock, USA; 2 Cardiology, Texas Tech University Health Sciences Center, Lubbock, USA

**Keywords:** left ventricular thrombus, cigarette smoking, aortic stenosis

## Abstract

The harmful effects of cigarette smoking on the human body have been well documented. However, whether tobacco use is an independent risk factor of valvular heart disease remains debatable. Cigarette smoking has been associated with an inflammatory state and increased levels of tumor necrosis factor alpha, which in turn activates protein kinases involved in ventricular remodeling. Subsequent ventricular dysfunction predisposes to the formation of mural thrombi which may lead to further worsening of hemodynamics. We present a case of severe aortic stenosis and giant left ventricular thrombus formation associated with chronic cigarette smoking.

## Introduction

Smoking kills approximately five million people per year worldwide. This accounts for over 20% of deaths in adult males, and 5% of deaths in adult females. More than a billion people are exposed to active or passive cigarette smoke around the world. Its harmful effects on the human body have been extensively researched and documented [1]. Cigarette smoking has been shown to induce oxidative stress, vascular inflammation, and platelet activation detrimental to the cardiovascular system [2]. We present a case of chronic cigarette smoking associated with severe aortic stenosis (AS) and a giant mural thrombus of the left ventricle (LV).

## Case presentation

**Figure 1:**
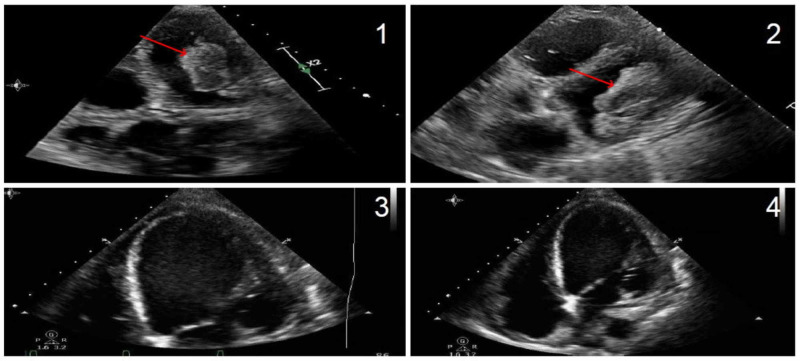
Echocardiogram demonstrating left ventricle thrombus Panels 1 and 2: Preoperative images (thrombus marked with arrows). Panels 3 and 4: Postthrombectomy images.

A 60-year-old female with a 105-pack year smoking history presented to the emergency department with three weeks of progressive chest discomfort and dyspnea on exertion. She was found to have mildly elevated cardiac biomarkers and a serum proB-type natriuretic peptide (proBNP) of 3,000 pg/ml. Transthoracic echocardiography (TTE) showed an LV ejection fraction (LVEF) of 20%-24%, severe global hypokinesis, severe AS with a mean pressure gradient of 21.9 mmHg, and a large bifid intracardiac LV mass, measuring 5 cm x 3.1 cm (Figure 1). Coronary angiography showed no significant coronary artery disease. After clinical stabilization, the patient underwent mechanical aortic valve replacement and thrombectomy in the same surgical session. The pathologic review of the mass tissue confirmed a large organizing thrombus. Postoperatively, the patient was started on anticoagulation with significant improvement in symptoms. Repeat TTE prior to discharge showed mildly improved LVEF of 25%-29%.

## Discussion

There are several studies that have indicated a strong association between myocardial infarction (MI) and cigarette smoking, with many describing nicotinic effects on cardiovascular dynamics. However, the understanding of any independent detrimental effect of tobacco use on heart valves remains limited. In the Cardiovascular Health Study and Multi-Ethnic Study of Atherosclerosis, current smoking status was linked to aortic valve disease [3]. Other investigators have also reported a relationship between aortic valve disease and active smoking [3,4]. Larsson et al. showed a strong correlation between cigarette smoking and AS [5]. Furthermore, Yamaura et al. showed associations between degenerative aortic valve disease and former smoking status, dose-dependent cigarette consumption, and a reduced odds ratio for the development of aortic valve disease after quitting tobacco use for at least 10 years [6]. However, there are several limitations in the study, including small sample size and poor generalizability, as the study population consisted of a small number of reasonably healthy men of working age.

The pathogenesis of AS shares many elements with that of atherosclerosis, including endothelial dysfunction, oxidized lipid transudation, inflammation, and proteolysis [7]. Studies have shown that calcific AS is associated with hypertension, dyslipidemia, elevated lipoprotein(a) levels, and diabetes [8]. Gu et al. found that cigarette smoking increases levels of norepinephrine and tumor necrosis factor alpha, which activates mitogen-activated protein kinases (MAPKs) and, ultimately, has been shown to cause ventricular remodeling and systolic dysfunction. It has been postulated that MAPKs play a major role in cellular growth and survival in the myocardium [9].

It is uncommon for cardiomyopathy and AS to develop in the setting of tobacco abuse without concurrent obstructive coronary artery disease, as was the case with our patient. The increased risk of thrombosis with cigarette smoking, particularly of the coronary circulation, is well documented in the literature [10]. Newby et al. demonstrated markedly impaired release of tissue plasminogen activator (tPA) in response to pharmaceutical-grade substance P infusion in smokers compared to nonsmokers, suggesting a mechanism by which smokers are more predisposed towards pathological clot formation [11]. However, the formation of cardiac mural thrombi in the absence of MI is uncommon, although a number of cases have been reported in patients with severe LV dysfunction [12]. LV thrombi can themselves compromise cardiac hemodynamics. Garg et al. used cardiovascular magnetic resonance (CMR) with kinetic energy (KE) flow acquisition and mapping to show that LV thrombi following MI significantly reduce and delay diastolic wash-in of the LV [13]. Other studies have shown that smoking can result in biventricular diastolic dysfunction [14].

It is plausible that the patient's chronic cigarette smoking contributed to the development of her severe AS, predisposing her to myocardial stress, resulting in combined systolic and diastolic heart failure, culminating in thrombus formation. Surgical thrombectomy should be performed on a case-by-case basis for patients with a large LV mural thrombus, depending on concurrent cardiac valvular and ischemic comorbidities, as most cases with LV thrombi in the literature were successfully treated with anticoagulation [15-20].

## Conclusions

This case of chronic cigarette smoking-associated severe AS and giant LV mural thrombus formation illustrates the possible association of cigarette smoking with thrombosis and cardiac valve pathology. Valvular pathology secondary to tobacco exposure leads to impaired hemodynamics, predisposing to mural thrombus formation in the setting of tobacco-induced prothrombotic state, which leads to a vicious cycle of progressive pump dysfunction and thrombus growth. It is important for physicians and patients alike to recognize the effects of cigarette smoking on the heart in order to take steps to break this cycle.
